# Blood purification with a cytokine adsorber for the elimination of myoglobin in critically ill patients with severe rhabdomyolysis

**DOI:** 10.1186/s13054-021-03468-x

**Published:** 2021-01-28

**Authors:** Christina Scharf, Uwe Liebchen, Michael Paal, Michael Irlbeck, Michael Zoller, Ines Schroeder

**Affiliations:** 1 Department of Anesthesiology, LMU Hospital, Marchioninistrasse 15, 81377 Munich, Germany; 2 Institute of Laboratory Medicine, LMU Hospital, Munich, Germany

**Keywords:** Cytosorb ®, Blood purification, Rhabdomyolysis, Myoglobin, Acute kidney injury

## Abstract

**Background:**

Rhabdomyolysis is frequently occurring in critically ill patients, resulting in a high risk of acute kidney injury (AKI) and potentially permanent kidney damage due to increased myoglobin levels. The extracorporeal elimination of myoglobin might be an approach to prevent AKI, but its molecular weight of 17 kDa complicates an elimination with conventional dialysis membranes. Question of interest is, if myoglobin can be successfully eliminated with the cytokine adsorber Cytosorb® (CS) integrated in a high-flux dialysis system.

**Methods:**

Patients were included between 10/2014 and 05/2020 in the study population if they had an anuric renal failure with the need of renal replacement therapy, if CS therapy was longer than 90 min and if myoglobin level was > 5.000 ng/ml before treatment. The measurement times of the laboratory values were: d-1 = 24–36 h before CS, d0 = shortly before starting CS and d1 = 12–24 h after starting CS treatment. Statistical analysis were performed with Spearman’s correlation coefficient, Wilcoxon test with associated samples and linear regression analysis.

**Results:**

Forty-three patients were included in the evaluation (median age: 56 years, 77% male patients, 32.6% ECMO therapy, median SAPS II: 80 points and in-hospital mortality: 67%). There was a significant equilateral correlation between creatine kinase (CK) and myoglobin at all measurement points. Furthermore, there was a significant reduction of myoglobin (*p* = 0.03, 95% confidence interval (CI): − 9030, − 908 ng/ml) during CS treatment, with a median relative reduction of 29%. A higher median reduction of 38% was seen in patients without ongoing rhabdomyolysis (CK decreased during CS treatment, *n* = 21). In contrast, myoglobin levels did not relevantly change in patients with increasing CK and therefore ongoing rhabdomyolysis (*n* = 22, median relative reduction 4%). Moreover, there was no significant difference in myoglobin elimination in patients with and without ECMO therapy.

**Conclusion:**

Blood purification with Cytosorb® during high-flux dialysis led to a significant reduction of myoglobin in patients with severe rhabdomyolysis. The effect might be obscured by sustained rhabdomyolysis, which was seen in patients with rising CK during treatment. Prospective clinical trials would be useful in investigating its benefits in avoiding permanent kidney damage.
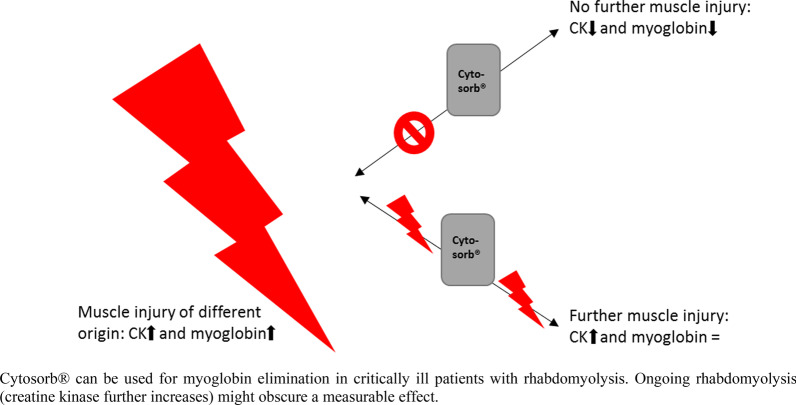

## Introduction

Rhabdomyolysis describes the disintegration of skeletal muscles, leading to the release of muscle components into the blood [1]. The most common cause is traumatic damage of the muscles, for example in patients after multiple traumas [2]. However, the clinical picture can also occur as part of severe sepsis and due to drug side effects [3]. Ultimately, many intensive care unit (ICU) patients are affected. In addition to electrolyte disorders caused by cell decay, there is also an accumulation of creatine kinase (CK) and myoglobin in the blood [4]. Even though there are no uniform diagnostic criteria, rhabdomyolysis is typically referred to when the CK is > 5 times the upper normal [5].

After muscle damage, CK reaches its maximum value in the blood after about 24 h and is then inactivated by oxidation in the blood independently of liver and kidney function [6]. Persistent muscle damage can further increase serum CK levels. Myoglobin reaches its maximum value in the blood within about 12 h after muscle damage and is rapidly eliminated by the kidneys. The half-life is about 3 h, if kidney function is unrestricted and muscle damage is not persistent [7]. However, an accumulation of large amounts of myoglobin might have nephrotoxic effects although a clear mechanism about the renal toxicity is still not clear. Thus, renal vasoconstriction, the formation of intratubular casts and direct toxic effect of myoglobin on the tubule cells might lead to acute kidney injury (AKI) and the clinical picture of the “crush kidney” [8].

A frequently used conservative treatment option in preventing AKI is the alkalization of the urine, but no randomized controlled study showed its benefits so far. However, renal replacement therapy (RRT) is necessary in many ICU patients due to ongoing AKI [9]. Although the amount of CK defines rhabdomyolysis, its elimination is of secondary importance due to the absence of toxicity [10]. In contrast, myoglobin can cause persistent and potentially permanent damage of the tubular epithelial cells in the kidney and its elimination from the blood might be an approach to avoid AKI [8]. The problem is that myoglobin, with a molecular weight of approximately 17 kDa, is only poorly removed by many standard dialysis membrane used in critically ill patients with the need of RRT [8, 11]. The surface area of the dialyzers is often between 1 and 2 m^2^ and the sieving coefficient for myoglobin mostly between 0.2 and 0.4.

One solution for a better myoglobin removal is the use of high cut-off (HCO) dialyzers. Heyne et al. [12] described this approach in a case series with an effective removal of myoglobin. Furthermore, Weidhase et al. [13] recently published the results of a randomized trial, where a higher myoglobin clearance can be seen in patients treated with HCO dialyzers in contrast to the typically used high-flux dialyzers. Both had the same surface area of 1.8 m^2^ and myoglobin clearance was about 8–10 ml/min for HCO dialyzers and 2–3 ml/min for high-flux dialyzers. Another method is plasmapheresis, where a successful myoglobin elimination had also been described [14]. A new approach is the use of the cytokine adsorbed Cytosorb® (CS) (CytoSorbents Europe, Berlin, Germany), which is primarily intended for the elimination of cytokines in hyperinflammatory conditions [15]. It has a surface area of about 45.000 m^2^ and hydrophobic molecules up to a size of approximately 55 kDa can be eliminated. The product has a CE mark for the elimination of myoglobin since 2019. Based on its molecular weight of 80 kDa, an elimination of CK cannot be assumed.

Although in vitro data showed the elimination of myoglobin by CS [16], there is a lack of clinical studies in the literature, except of two case reports. [17, 18]. Due to the lack of data describing the effects of CS in patients with rhabdomyolysis, a retrospective data analysis was investigated in critically ill patients with severe rhabdomyolysis and CS therapy. Within the scope of clinical practice, CS was used at two anesthesiological ICUs at the LMU hospital in Munich in patients with severe rhabdomyolysis and the need of RRT. To evaluate the clinical experience in a structured way and to gain first results on the *in-vivo* efficacy of CS in critically ill patients with rhabdomyolysis, a retrospective data analysis was performed.

## Methods

### Study setting

This was a single-center, retrospective observational study investigating the effects of CS therapy in critically ill patients with AKI and rhabdomyolysis. Patients were included between October 2014 and May 2020 during their stay at two ICUs at the LMU hospital in Munich. The local institutional review board approved the study (registration number 20-477).

### Laboratory measurements and data collection

All clinical–chemical parameters were determined with standard clinical chemistry tests in the institute of laboratory medicine. CK was measured using a kinetic test, so all different isoforms are included. Myoglobin was measured with a muscular specific immunoassay. Demographic data, clinical variables and laboratory variables were collected from the laboratory and patient information system. Baseline characteristics (age, gender, body mass index (BMI), extracorporeal membrane oxygenation (ECMO), days at ICU till treatment with CS, 7-days mortality, in-hospital mortality, sequential organ failure assessment (SOFA) score, Simplified Acute Physiology Score (SAPS) II, reason for admission to ICU and reason for rhabdomyolysis) were evaluated on the treatment day. Laboratory data (CK, myoglobin and lactate serum levels) were collected daily before and during the intervention.

### Study population

All patients, who were treated with CS between 10/2014 and 05/2020, were screened for evaluation. Patients were only included in the study population when CS therapy was longer than 90 min and it was the first treatment interval. Furthermore, myoglobin serum level had to be over 5000 ng/ml, measured directly before CS treatment. Furthermore, a myoglobin level had to be measured directly after CS therapy. A repetitive application took place partially, but was excluded in our evaluation. Moreover, only patients with anuria and the need of continuous RRT with high-flux dialysis (Fresenius Ultraflux® AV 600S, surface area 1.4 m^2^, myoglobin sieving coefficient ~ 0.3) were included.

### Blood sampling

In the data evaluation, three time points were considered depending on CS treatment:

- d-1: 24–36 h before starting CS therapy.
- d0: 0–12 h before starting CS therapy (= directly before treatment).
- d1: 12–24 h after starting CS therapy (= end of treatment or immediately after the end).

### Statistical analysis

Statistical analysis was performed with IBM SPSS statistics (Version 26.0. IBM Corp., Armonk, NY, USA). The effect of CS treatment on the reduction of myoglobin in relation to CK was investigated. Continuous variables are given as median and interquartile range (IQR) or with minimum and maximum. The relative change of the parameters (%) was calculated with: 100 − ((100/parameter d0) * parameter d1). As laboratory variables had no normal distribution in the Shapiro–Wilk test, nonparametric tests were used. Spearman’s correlation coefficient was used for correlation analysis and Wilcoxon test with associated samples was used to describe the change of CK and myoglobin before and during CS treatment. To identify confounders on the effect of myoglobin elimination with CS (relative change of myoglobin during CS therapy), a linear regression analysis was performed. Independent variables were: relative change of CK (%) (d0–d1), CK d0, myoglobin d0, lactate d0, relative change of lactate (%) (d0–d1), SAPS II d0 and ECMO therapy. To identify baseline changes and changes in the elimination of myoglobin in patients with and without ECMO-therapy, the *U*-test was used.

## Results

### Demographic and clinical data

In total, 43 patients were included in the evaluation. CS was integrated into a continuous renal replacement procedure as all patients had an AKI requiring RRT. The main diagnoses at admission to the ICU were as follows, in descending order: acute respiratory distress syndrome (ARDS) (30.2%), polytrauma (18.6%), septic shock (14.0%) and solid organ transplantation (14.0%). The reason for rhabdomyolysis was as follows, in descending order: infection/septic shock (41.9%), trauma (23.2%), hypovolemic or cardiogenic shock (14.0%), metformin intoxication (2.3%) and unknown origin (18.6%). The median age was 56 years, 77% were male and 32.6% needed support with ECMO therapy. All patients were critically ill, which was reflected in a median SAPS II of 80 points, which predicted a mortality rate of 92.5%. The actual in-hospital mortality rate was 67.4%. Detailed patient characteristics can be found in Table 1.

**Table 1:** Patient characteristics and laboratory measurements

	n (%) or median [Range: min, max]
Patient characteristics	
Age (years)	56 [18, 84]
Gender: male/female	33 (76.7)/10 (23.3)
BMI (kg/m^2^)	26.7 [15.2, 45.0]
ECMO therapy	14 (32.6)
Days at ICU till treatment	2 [0, 30]
7-day mortality	11 (25.6)
In-hospital mortality	29 (67.4)
SOFA score on treatment day	19 [8, 23]
SAPS II on treatment day	80 [40, 115]
Laboratory measurements	
Myoglobin d-1 (ng/ml)	9938 [1253, 260,000]
Myoglobin d0 (ng/ml)	25,281 [7033, 252,000]
Myoglobin d1 (ng/ml)	22,871 [3548, 163,000]
CK d-1 (U/l)	2389 [96, 33,643]
CK d0 (U/l)	6752 [855, 200,000]
CK d1 (U/l)	6652 [822, 90,350]

*BMI* body mass index, *ECMO* extracorporeal membrane oxygenation, *ICU* intensive care unit, *SOFA* sequential organ failure assessment, *SAPS* Simplified Acute Physiology Score, *CK* creatine kinase, *d* day

### Comparison of CK and myoglobin levels before and after Cytosorb® treatment

The laboratory parameters were examined at d-1, d0 and d1 as described in the method section. Furthermore, the relative change (%) during the time periods (d-1/d0) and (d0/d1) for both CK and myoglobin was analyzed. Spearman’s correlation coefficient showed a significant, moderate equilateral correlation in the comparison of CK and myoglobin at all time points.

**Fig. 1:**
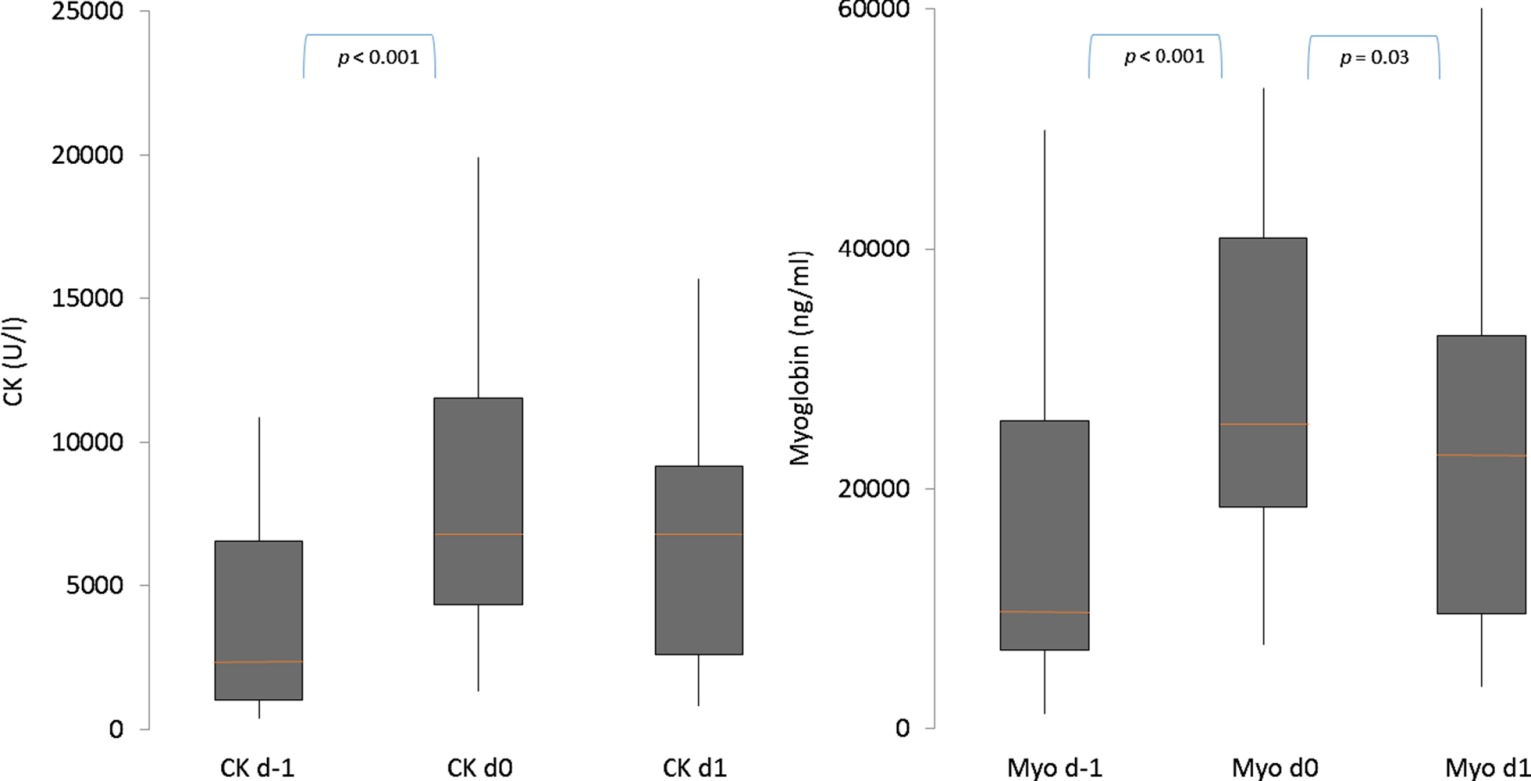
CK and myoglobin values of all patients at different time points. *Myo* Myoglobin, *CK* creatine kinase, *d-1* 24–36 h before Cytosorb® treatment, *d0* 0–12 h before CS treatment, *d1* 12–24 h after beginning Cytosorb® treatment, orange line represents the median, gray boxes the interquartile range and the whiskers are limited to 1.5 times the interquartile range

Statistical analysis with the Wilcoxon test with associated samples showed a significant increase of CK in the period prior to CS treatment *(p* < 0.001, CI 2287, 5923 U/l) with a median relative change of + 129.2% (IQR: 45.8%, 310.1%). It was the same for myoglobin prior to CS therapy (*p* < 0.001, CI 8774, 20,173 ng/ml) with a median relative change of + 106.1% (IQR: 40.6%, 213.1%). There was no significant change of CK during CS therapy (*p* = 0.419, CI − 1542, 669 U/l; median relative change + 1.0% (IQR: − 28.3, + 78.2%)). In contrast, a significant reduction of myoglobin can be seen during CS treatment (*p* = 0.03, CI − 9030, − 908 ng/ml) with a median relative change of − 29.4% (IQR: − 41.2, + 2.6%). Figure 1 shows the course of CK and myoglobin prior and during CS therapy as box plots.

A regression analysis was performed to detect determinants that affect the change of myoglobin during CS therapy. The only significant factor that influenced the relative change of myoglobin was the relative change of CK during the same period (*p* < 0.001, CI 0.39, 0.61). All other parameters had no significant impact on the relative change of myoglobin during CS treatment.

### Effect of Cytosorb® in different groups

**Fig. 2:**
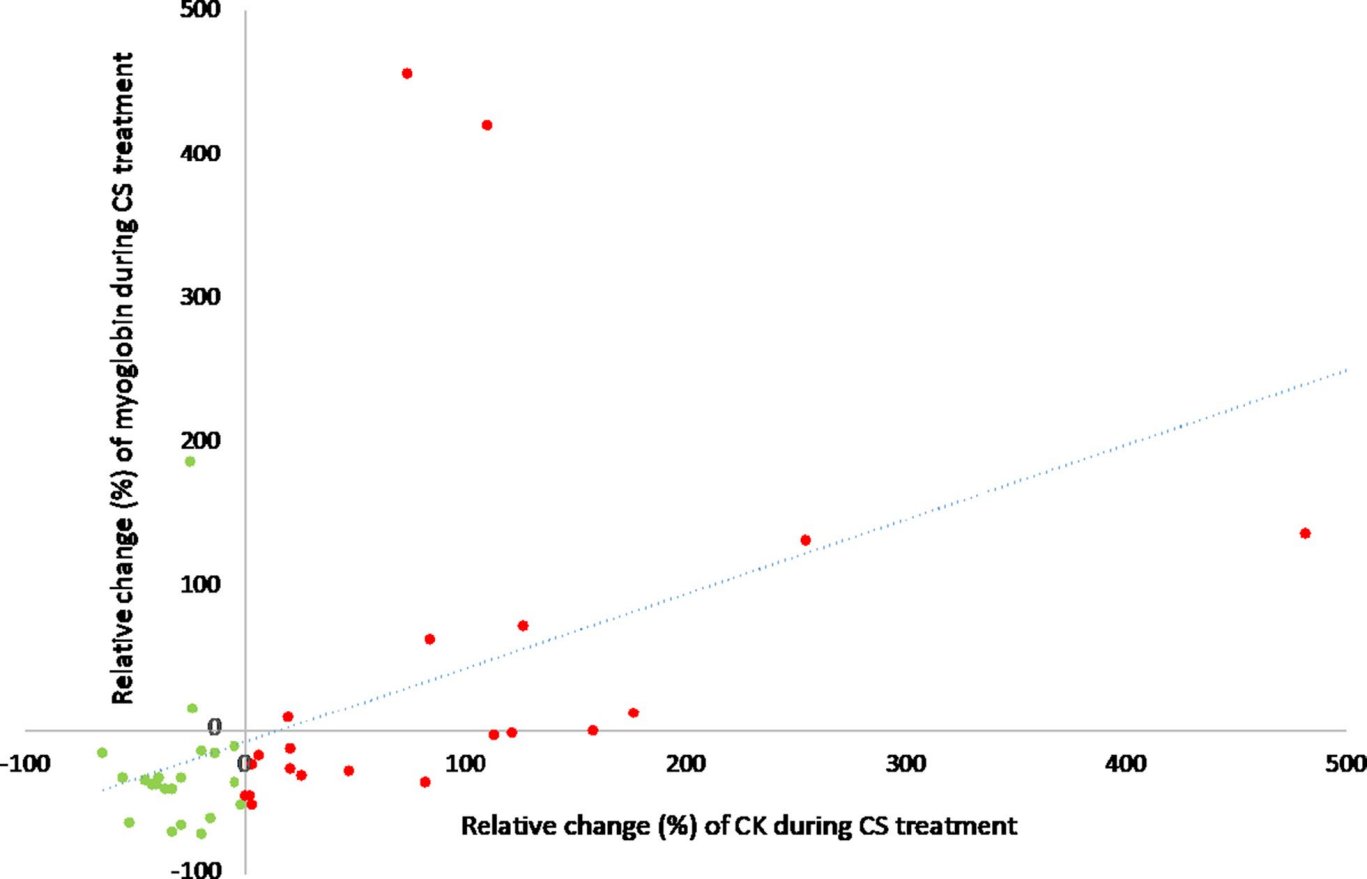
Relative change (%) of myoglobin and CK during CS treatment. *CK* creatine kinase, *CS* Cytosorb®, green dots: decrease of CK during CS treatment (= group 1), red dots: increase of CK during CS treatment (= group 2)

We assumed that myoglobin elimination by CS therapy was not visible in laboratory measurement due to persistent rhabdomyolysis with accumulation of further myoglobin and CK. Therefore, patients were divided into two groups depending on whether CK increased or decreased during CS application. Group 1 (*n* = 21) was defined as patients with decreasing CK during CS and group 2 (*n* = 22) was defined as patients with increasing CK, which is equal to an ongoing muscle injury. Figure 2 shows the relative change (= 100 − ((100/parameter d0) * parameter d1) of myoglobin und CK during CS treatment. Patients allocated to group 1 are presented as green dots and patients allocated to group 2 as red dots.

The periods prior to CS (d-1/d0) and the period during CS (d0/d1) were analyzed concerning CK and myoglobin levels and relative change in the two different groups. Detailed results can be found in Table 2.

**Table 2:** CK and myoglobin in the two different groups

CK	Median (IQR) or relative change [IQR]	Myoglobin	Median (IQR) or relative change [IQR]
Group 1 (decreasing CK)			
CK d-1	2715 (887, 6392)	Myoglobin d-1	16,417 (6474, 25,622)
CK d0	6982 (4605, 9903)	Myoglobin d0	29,514 (20,744, 38,317)
CK d1	4962 (2802, 7946)	Myoglobin d1	19,355 (10,481, 29,667)
100 − ((100/CK d-1) * CK d0)	132% [29, 513]	100 − ((100/Myo d-1) * Myo d0)	73% [37, 132]
100 − ((100/CK d0) * CK d1)	− 28% [− 39, − 19]	100 − ((100/Myo d0) * Myo d1)	− 38% [− 53, − 18]
Group 2 (increasing CK)			
CK d-1	1833 (776, 4011)	Myoglobin d-1	9406 (5081, 10,701)
CK d0	4178 (2232, 11,731)	Myoglobin d0	23,264 (12,956, 27,769)
CK d1	11,819 (5112, 20,193)	Myoglobin d1	24,297 (14,044, 53,745)
100 − ((100/CK d-1) * CK d0)	126% [79, 283]	100 − ((100/Myo d-1) * Myo d0)	166% [97, 351]
100 − ((100/CK d0) * CK d1)	78% [21, 126]	100 − ((100/Myo d0) * Myo d1)	− 4% [− 30, 69]

*CK* creatine kinase, *Myo* myoglobin

There was a significant increase of CK (*p* = 0.025, CI 1495, 19,168 U/l) and myoglobin (*p* = 0.01, CI 6749, 37442 ng/ml) in patients allocated to group 1 prior to CS treatment. Moreover, a significant decrease of both CK (*p* < 0.001, CI − 3777, − 1168 U/l) and myoglobin (*p* < 0.001, − 19,386, − 7111 ng/ml) can be seen. The median relative reduction of myoglobin was 38%.

In contrast, there was no significant change, neither for CK (*p* = 0.201, CI − 2478, 8898 U/l), nor for myoglobin (*p* = 0.147, CI − 6472, 19,502 ng/ml) prior to CS treatment in patients allocated to group 2. During treatment with CS, a significant increase in CK (*p* < 0.001, CI 1861, 6946 U/l) was observed in those patients, while there was no significant change of myoglobin (*p* = 0.858, CI − 4954, 29,770 ng/ml).

**Fig. 3:**
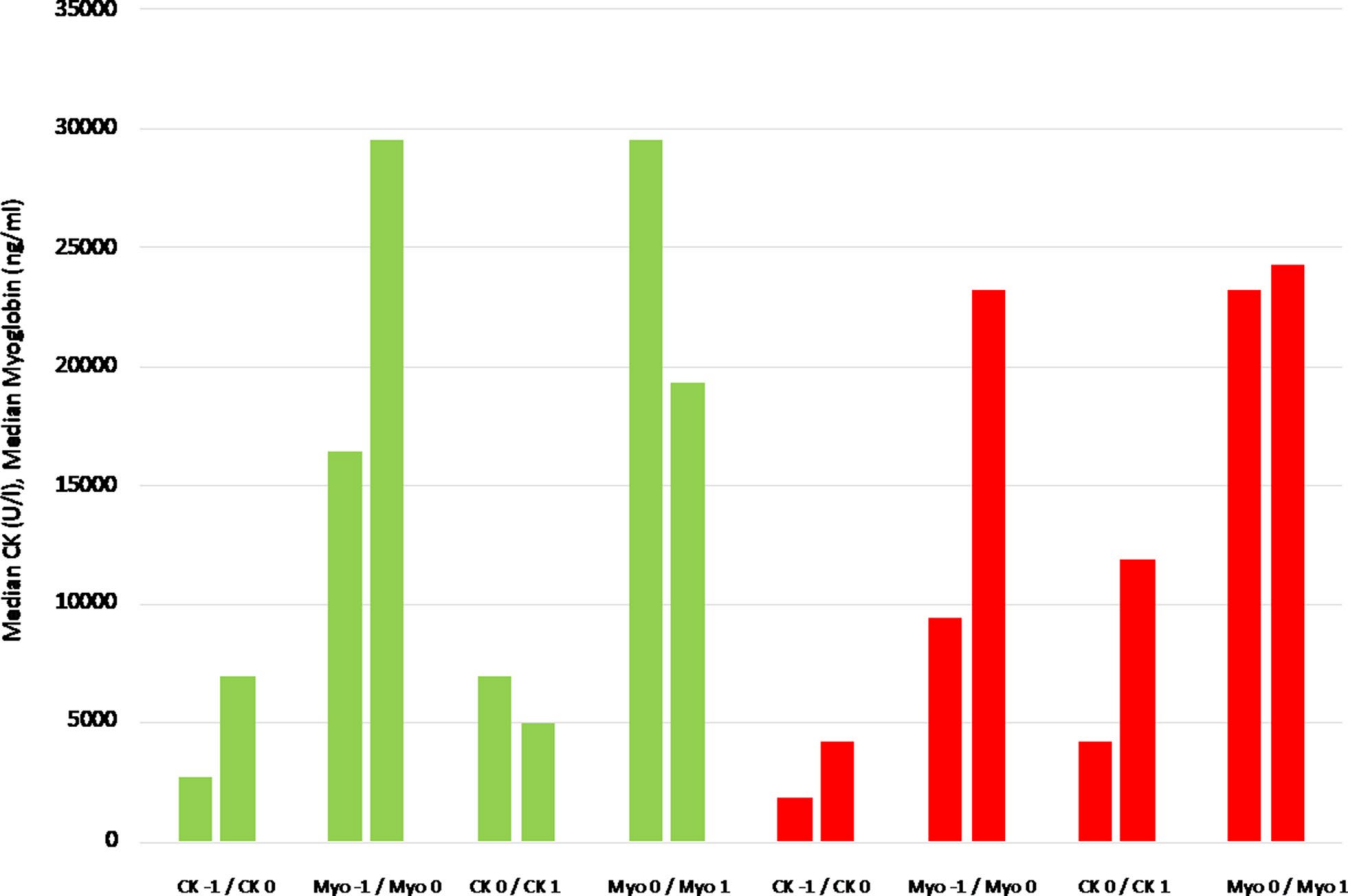
Median CK and myoglobin levels in the different groups. *CK* creatine kinase, *Myo* myoglobin, − 1: day − 1, 0: day 0, 1: day 1, green beams: decrease of CK during CS treatment (= group 1), red beams: increase of CK during CS treatment (= group 2)

Figure 3 shows the median CK and myoglobin levels in the different groups. The course of CK and myoglobin was equal in the observed period in patients allocated to group 1. It was the same in patients allocated to group 2 prior to CS therapy with increasing CK and myoglobin levels. In contrast, CK serum levels increased rapidly during CS treatment, while myoglobin serum levels remained nearly unchanged.

### Myoglobin elimination in patients with and without ECMO therapy

In total, 14 patients needed support with ECMO therapy and had therefore a high risk for hemolysis. The median LDH (U/l) at d0 in patients with and without ECMO therapy was 1566 (IQR: 767, 3824) and 825 (IQR: 690, 3190) with no significant difference in the *U*-test (*p* = 0.50). Furthermore, there was no difference in median myoglobin d0 (ng/ml) with 26,760 (IQR: 17,318, 35,311) and 25,012 (12,587, 37,562) in patients with and without ECMO-therapy (*p* = 0.88), respectively. The median relative reduction during CS treatment was 26.2% in ECMO-patients and 34.4% in patients without ECMO-therapy. A significant difference was also here not to be recognized (*p* = 0.31).

## Discussion

To the best of the authors' knowledge, this is the first analysis of the effectiveness of the cytokine adsorber Cytosorb® integrated into a continuous high-flux dialysis system for myoglobin elimination in critically ill patients with severe rhabdomyolysis, apart from case reports. With 43 patients, the study represents the largest population in which blood purification was used for myoglobin elimination. A SAPS II of 80 points was indicative for the severity of disease of the study population. A median myoglobin level of > 25,000 ng/ml before the start of CS therapy underlines the massive rhabdomyolysis. Although all conservative therapeutic options should be exhausted to avoid a crush kidney [9, 19], acute renal failure can usually not be avoided, especially at such high myoglobin concentrations.

Since extracorporeal myoglobin elimination is only insufficiently possible with high-flux dialysis (small surface area and low sieving coefficient), several special elimination procedures are available. As all patients had an anuric renal failure, the authors do not assume a relevant endogenous myoglobin elimination in the study population. The median myoglobin clearance of a similar high-flux filter was recently described by Weidhase et al. [13] with approximately 2–3 ml/min. However, a new alternative for myoglobin elimination might be CS, whose integration into an, for example, existing high-flux dialysis system is quick and easy. Even though there is regular approval for the usage of CS in patients with rhabdomyolysis in the European Union, there is a general lack of clinical studies [16]. It is important to know which molecules can theoretically be eliminated by CS based on their molecular size. The cutoff value of CS based on manufacturer’s specifications is about 55 kDa. Therefore, the elimination of myoglobin with a molecular size of 17 kDa is possible [20], whereas CK with a size of about 80 kDa cannot be eliminated [21]. With this assumption, the elimination of CK by CS described by Dilken et al*.* [18] must be critically questioned and is most likely based on the endogenous decay and not on removal by CS. This was also clearly stated by Daum et al*.* [22], who referred directly to the case report presented by Dilken et al*.*

A median relative reduction rate of 29% is a great success, especially with persistent rhabdomyolysis. Ongoing rhabdomyolysis might be the reason, why the effect of CS is often difficult to detect in laboratory measurements. However, correlation analysis revealed that there was a significant correlation between the CK and myoglobin values, and regression analysis showed that the change in CK during CS was the only factor that influenced myoglobin levels. Therefore, the two variables seem comparable, even though the flooding time and half-life of the substances are different [23].

Due to organ-independent degradation, there should be a relevant reduction of CK during CS application, assuming an expired rhabdomyolysis. In contrast, if the CK continues to increase, there might be an ongoing rhabdomyolysis. Therefore, two groups were formed based on this assumption. In conclusion, a significant reduction of myoglobin (median relative reduction: 38%) was detected in patients with decreasing CK (group 1). Since all patients had an anuric renal failure, renal elimination of myoglobin cannot be assumed and, in the authors' view, the reduction of myoglobin was largely due to the use of CS and not due to the high-flux dialyzer. The elimination rate was comparable to a study in pigs, where a myoglobin elimination of 41% by CS was described [24]. However, it should be noted that degradation pathways with spleen and liver have been rarely described [25].

Although no significant change in myoglobin was observed in patients with increasing CK (group 2), the elimination was evident from the substantial increase of CK during CS application (median relative increase of 78%), while there was a median decrease of 4% for myoglobin. Without CS therapy, a rapid increase in myoglobin, equivalent to the previously unidirectional change in CK and myoglobin, would be expected in those patients as well. Although the absence of an increase did not prove that CS therapy was successful, this must be supposed from the authors’ perspective.

As one-third of our patients needed support with ECMO-therapy, which potentially influences the performance of CS, a subgroup analysis was performed. Neither a more pronounced hemolysis, nor higher myoglobin levels before treatment or a significant difference in the relative myoglobin reduction can be seen. Although our population is not generally applicable to all other ICU patients, we conclude that ECMO therapy might not have relevant impact on CS elimination performance.

No clinical studies that examine the evidence of CS in the context of rhabdomyolysis are available. It further remains unclear whether and how much myoglobin elimination leads to less kidney damage and faster completion of dialysis. In this context, our study can provide the foundation for future prospective projects. Overall, other blood purification systems can also be used for the elimination of myoglobin [12, 26–29]. The advantages of CS compared to, for example, plasmaphereses are that albumin, with a molecular size of 66–67 kDa, is not absorbed and that it can be easily and temporarily integrated into an existing dialysis system. Ultimately, all procedures lack both individual studies and comparative studies questioning the clinical relevance of myoglobin elimination. Therefore, a final evaluation according to the best procedure is not possible.

In summary, myoglobin removal with the cytokine adsorber CS integrated into a high-flux dialyzer can be recommended for clinical routine due to its existing CE mark, ease of use and absence of side effects. Even though an advantage in patient outcome has not yet been confirmed, extracorporeal elimination seems to be useful especially in cases of high myoglobin levels and existing AKI. Therefore, potentially permanent damage of the renal tubules cells caused by the accumulation of myoglobin could be prevented [30]. Nevertheless, prospective studies are lacking in which the myoglobin clearance of CS can be accurately determined by a dense blood collection scheme and, for example, simultaneous measurements at the adorber’s in—and outlet, to determine cross-adsorber clearance.

Finally, this study has several limitations. First, it was a retrospective, single-center data analysis, whereby laboratory parameters were collected in clinical routine. Although the time of blood collection and the duration of treatment with CS were electronically documented, a slight deviation was still possible. Second, there was no control group, so the course of myoglobin without CS could not be assessed and elimination outside the kidneys may have played a minor role. In this context, a statement on the difference in patients’ outcome with and without CS treatment is not possible. Third, CK was measured using a kinetic test, so the CK value included all isoforms. Last, statements about the myoglobin clearance and saturation kinetics of the CS absorber are not possible due to the relatively sparse blood sampling scheme. Moreover, small amounts of myoglobin can also be eliminated by the high-flux dialysis and the elimination was therefore a combination of CS therapy and high-flux dialysis.

## Conclusion

Blood purification with Cytosorb® integrated into a high-flux dialyzer may be a useful tool for the elimination of myoglobin in critically ill patients with rhabdomyolysis. Myoglobin elimination could avert permanent kidney damage by avoiding its deposition in the kidney. A measurable effect might be obscured during sustained rhabdomyolysis, seen in patients with a rising CK during treatment. Thus, in-vivo pre- and post-adsorber measurements would be useful for investigating the elimination rate and elimination kinetic of myoglobin during CS treatment. Prospective randomized trials would, furthermore, be useful to examine the clinical benefits in terms of recovery of kidney function and patient outcome.

## Data Availability

All data generated during this study are included in this article.

## Abbreviations

AKI: Acute kidney injury
BMI: Body mass index
CI: Confidence interval
CK: Creatine kinase
ECMO: Extracorporeal membrane oxygenation
HCO: High cut off
ICU: Intensive care unit
RRT: Renal replacement therapy
SAPS: Simplified Acute Physiology Score
SOFA: Sequential organ failure assessment
